# The cellular kinetics of lung alveolar epithelial cells and its relationship with lung tissue repair after acute lung injury

**DOI:** 10.1186/s12931-016-0480-y

**Published:** 2016-12-07

**Authors:** Ling Zeng, Xue-tao Yang, Hai-sheng Li, Yong Li, Ce Yang, Wei Gu, Yin-han Zhou, Juan Du, Hai-yan Wang, Jian-hui Sun, Da-lin Wen, Jian-xin Jiang

**Affiliations:** 1 State Key Laboratory of Trauma, Burns and Combined Injury, Institute of Surgery Research, Daping Hospital, Third Military Medical University, Changjiang Road 10, Yuzhong District, 400042 Daping Chongqing, China; 2 Bristol Myers Squibb, Rewood City, CA USA

**Keywords:** Acute lung injury, Cellular kinetics, Regeneration, Type 1 alveolar epithelial cells, Type 2 alveolar epithelial cells

## Abstract

**Background:**

Organ regeneration in mammals is hypothesized to require a functional pool of stem or progenitor cells, but the role of these cells in lung regeneration is unknown.

**Methods:**

Based on the fact that postnatal regeneration of alveolar tissue has been attributed to alveolar epithelial cells, we established a hemorrhagic shock and Lipopolysaccharide (LPS) lung injury model. Using this model, we analyzed the cellular kinetics of lung alveolar epithelial cells.

**Results:**

The results showed that alveolar epithelium type 2 cells (AEC2s) are damage resistant during acute lung injury, they might be the main cells involved in lung injury and repair. Then we observed the relationship between the expression of HGF, c-Met following ALI in rat lung and proliferation of AEC2s. The proliferation of AEC2s was inhibited when isolated primary AEC2s were co-cultured with c-Met inhibitor SU11274. Furthermore, the numbers of AEC2s was significantly decreased when ALI rats were administrated with SU11274 in vivo. It provided further evidence that the HGF/c-Met signaling plays a vital role in ALI-induced AEC2s proliferation.

**Conclusions:**

AEC2s are damage resistant during acute lung injury and the HGF/c-Met signaling pathway is of vital importance in the proliferation of AEC2s after ALI.

## Background

Lung is a highly quiescent tissue, particularly, compared with other adult organs such as the intestine and liver. But now, it’s widely accepted that lung has a remarkable reparative capacity [1–3]. When the epithelial cells lining the interior of the lung are damaged by infection with influenza virus, a rare stem-cell population distal airway stem cells (DASCs) is induced to proliferate and migrate to the damaged site. They can differentiate into alveolar epithelium type 1 and type 2 cells (AEC1/2 s) [4]. Bronchioalveolar stem cell (BASCs) is a regional pulmonary stem cell population, identified at the bronchioalveolar duct junction [5]. It is resistant to bronchiolar and alveolar damage and proliferate during epithelial cell renewal in vivo [6]. Vaughan et al proposed a lineage-negative epithelial progenitor (LNEP) as the major source of induced Krt5+ cells and it mobilize to regenerate lung epithelium after major injury [7]. Using a microfluidic magnetic activated cell sorting system, our previous study has isolated mouse lung multipotent stem cells (MLSCs) which play an important role in bronchiolar and alveolar epithelial cells injury repair [8]. To our interest, BASCs, DASCs, LNEP and MLSCs are all rare stem cells which play their role in regeneration through differentiation into lung progenitor cells, especially AEC2s.

AEC2s are widely accepted as progenitor cells of lung and contribute to the lung repair and regeneration process. During development, AEC1s and AEC2s arise from a bipotent progenitor cell lineage, whereas after birth, AEC2s can undergo long-term self-renewal and give rise to AEC1s during homeostasis [9, 10]. But the mechanisms that regulate AEC2 renewal are incompletely understood. More recently, genetic lineage tracing experiments showed evidence that AEC2s were capable of long-term self-renewal and the generation of AEC1 in both alveolar regeneration and homeostasis. Therefore AEC2s are stem cells, as a population, proliferate in vivo and give rise to AEC1s [11, 12].

Acute lung injury (ALI) and Acute Respiratory Distress Syndrome (ARDS) are frequently seen in traumatically injured patients. They remain significant contributing factors to morbidity in the traumatically injured patient. Survivors of ARDS often have a lower functional ability and lower than normal health related quality of life [13]. During the course of ALI, multi-factors such as the activation of inflammatory cells and release of inflammatory factors lead to damage of air-blood barrier (ABB). However, present treatments such as infection control and mechanical ventilation are supportive therapies, promoting the regeneration of lung itself maybe an ideal therapy. In this study, we established a hemorrhagic shock and LPS lung injury model. Using this model, we analyzed the cellular kinetics of lung alveolar epithelial cells. In terms of mechanism research, we explored the role of HGF/c-Met signaling pathway in AEC2s proliferation after ALI. Accordingly, we first examined the proliferation of AEC2s, expression of HGF after ALI, and phosphorylation of c-Met following ALI in rat lung. Western blotting using the c-Met inhibitor SU11274 provided further evidence for the involvement of HGF/c-Met signaling in ALI-induced AEC2s proliferation.

## Methods

### Hemorrhagic shock and LPS lung injury model

Male Sprague-Dawley rats, 220–250 g, were anaesthetized with pentobarbital sodium (60 mg/kg). Immediately after induction of anaesthesia. We inserted a catheter into the femoral artery and registered the blood pressure. Then, the blood pressure was decreased to 35–40 mmHg in 10 min and lasted for 1.5 h by drawing blood from the femoral artery. After recovery, LPS (4.0–7.0 mg/kg) were instilled intratracheally in 200ul phosphate-buffered saline (PBS). The sham operation control group was given femoral artery cannulation but without hemorrhagic shock and LPS instillation. SU11274 treated group was administrated with SU11274 (10 mg/kg) by intraperitoneal injection for 7 consecutive days post ALI. Rats were sacrificed on days 1, 2, 3, 5 and 7 after injury to study time-specific proliferation of lung epithelial cells (*n* = 10 for each group). Animals were purchased from SPF Laboratory Animal Room (Chongqing, China). The rats were housed in a temperature and humidity-controlled, pathogen-free facility with a 12 h light-dark cycle (12 L: 12 D). The Institutional Animal Care and Use Committee of the Institute of Zoology, the Third Millitary Medical University approved all the procedures. All experiments were performed in accordance with the Institutional Animal Care and Use Committee guidelines.

### Immunofluorescence

Five-micrometer sections of adult rat lung were fixed with paraformaldehyde (4%) and embedded in optimal cutting temperature medium were incubated in blocking buffer (1 h, 5% wt/vol BSA, 1% skim milk, 0.05% Triton X-100 in PBS). Sections were then incubated overnight with rabbit anti-proSPC antibody (Millipore) or mouse monoclonal PCNA (Abcam). And then washed in PBS (0.05% Tween 20). Sections were washed and then incubated with donkey anti- rabbit or anti-mouse conjugated to Alexafluor 568 (Invitrogen) and Alexafluor 488-labeled tyramide (Invitrogen) for 1 h and then washed. Nuclei were stained with DAPI, followed by rinsing and mounting in Vectashield mounting medium (Vecta Laboratories).

### Stereological analysis

For stereological analysis, 5 random lung tissue sections stained by HE were observed from every lobe (4 lobes in the right lung and 1 lobe in the left) in each rat lung tissue (5 animals per group). The area of photograph is non-hemorrhage area adjacent to the hemorrhage area. The number of pro-SPC positive cells was determined by counting positively stained cells per high power field (magnification X 600). Five even distributed areas of stained lung sections from five blocks of lung tissue in each group (3 animals per group) were counted. Nine consecutive images were taken in each area. Cell numbers in Fig. 2d are the mean ± SD for AT2 cell numbers per high power field (The area of the field at this magnification was 0.018 mm^2^) in sham group, post acute lung injury group and SU11274 treated group. Alveolar epithelial cells were identified by pro-SPC positive staining (AEC2s) and AQP5 positive staining (AEC1s). Proliferated cells were identified by PCNA positive staining. The length of basement membrane of alveoli was measured by Image J (version 2.0).

### Flow cytometry analysis

The right lower lobes of right lung were prepared for flow cytometry analysis. In brief, 5 ml dispase I (10U/mL, BD) was injected through the bronchi. Subsequently, the lungs were incubated in a 37 °C shaking incubator for 45 min in 10 mL of dispase(10U/mL), 1 mL of 0.001% DNAse (Sigma), and 1 mL of 2 μg/mL collagenase/dispase (Roche). The bronchi were removed, and the lungs were minced and incubated for 5 min. This suspension was filtered by 35 μM filter, centrifuged, and depleted of red blood cells by incubation in RBC lysis buffer (Sigma). Primary antibodies including rabbit anti-proSPC, rabbit anti-AQP5 were added to incubate cells. These antibodies were detected following incubation with FITC conjugated donkey anti-rabbit. Dead cells were discriminated by 7-Amino-Actinomycin D (7-AAD) staining.

### Western blot

Tissue or cells were lysed in lysis buffer (10 mM Tris-HCl, pH 7.5, 1% Triton X-100, 1 mM EDTA, and 1 mM phenylmethylsulfonyl fluoride, 10 g/ml aprotonin, and 10 g/ml leupeptin). The protein concentration was determined by the BCA protein assay kit (GenStar, Beijing, China). 30 ug of protein was separated on 12% SDS-polyacrylamide gels, transferred to a nitrocellulose membrane using the semidry transfer apparatus (Bio-Rad) at 17 mA for 60 min. The membrane was stained with Ponceau S to ensure proper transfer and blocked overnight with 5% dry skim milk powder in 100 mM Tris-buffered saline plus 0.1% Tween 20 (TBS-T). The membranes were incubated with antibodies overnight at 4 °C. After being washed in TBS-T 3 times, the membranes were incubated with horseradish peroxidase-conjugated anti-mouse, -goat, or -rabbit IgGs (1:400) for 1 h. The blots were washed again. The individual target proteins were visualized using the enhanced chemilumi-nescence detection system.

### ELISA

Vascular Endothelial Growth Factor (VEGF), Epidermal Growth Factor (EGF), Keratinocyte Growth Factor (KGF) and Hepatocyte Growth Factor (HGF) in the lung homogenate from acute lung injury were detected by Sandwich Enzyme Linked Immunosorbent assay (ELISA), according to the manufacturer’s instructions (Takara, Japan). The detection limits of the assay were 4 pg/ml.

### Cell isolation and culture

We optimized a protocol for isolating alveolar epithelial cells on the basis of immunomagnetic enrichment. The isolation mainly includes two parts. First, rat IgG panning to deplete immunocytes expressing FcR to enrich for alveolar epithelial cells. Second, immunomagnetic capture using magnetic beads conjugated to monoclonal antibody against specific membrane markers-T1α (Sigma, USA) to purify AEC1s and EpCAM (Abcam, USA) to purify AEC2s.

The pneumocytes plate was subjected to MACS immunomagnetic separation according to the manufacturer’s specifications (Miltenyi Biotec). Briefly, cells were incubated with rabbit anti-rat T1α antibodies (Sigma, USA) for 40 min at 4 °C. Cells were then incubated with goat anti-rabbit Micro-Bead solution at 4 °C for 15 min. Then they were centrifuged for 5 min and resuspended with 1 ml of the separation buffer. The cell suspension was applied on a MACS separation column subjected to a magnetic field provided by the MACS separator. The column was washed three times with 500 μl separation buffer and then released from the magnetic field, allowing the T1α-expressing cells to be eluted into a separate tube. The isolated T1α-expressing epithelial cells were termed AEC1s. To gain high purity of AEC2s, collect T1α-negative cells, incubated with mouse anti-rat EpCAM antibodies (Abcam, USA) for 40 min at 4 °C, and then handle cells as above with rat anti-mouse MicroBead solution. Thereafter isolated EpCAM-expressing epithelial cells were AEC2s. Sorted AEC2 cells were cultured with Dulbecco’s Modified Eagle’s Medium/10% FBS/penicillin/streptomycin.

### Statistical analysis

The results are presented as mean ± SEM; statistical analysis was performed using either one-way analysis of variance followed by Student-Newman-Keuls multiple comparisons post-hoc analysis or Kaplan-Meier survival analysis as appropriate, with a p value of less than 0.05 considered significant.

## Results

### Kinetics of alveolar epithelial cells after acute lung injury

#### Acute lung injury model

**Fig. 1:**
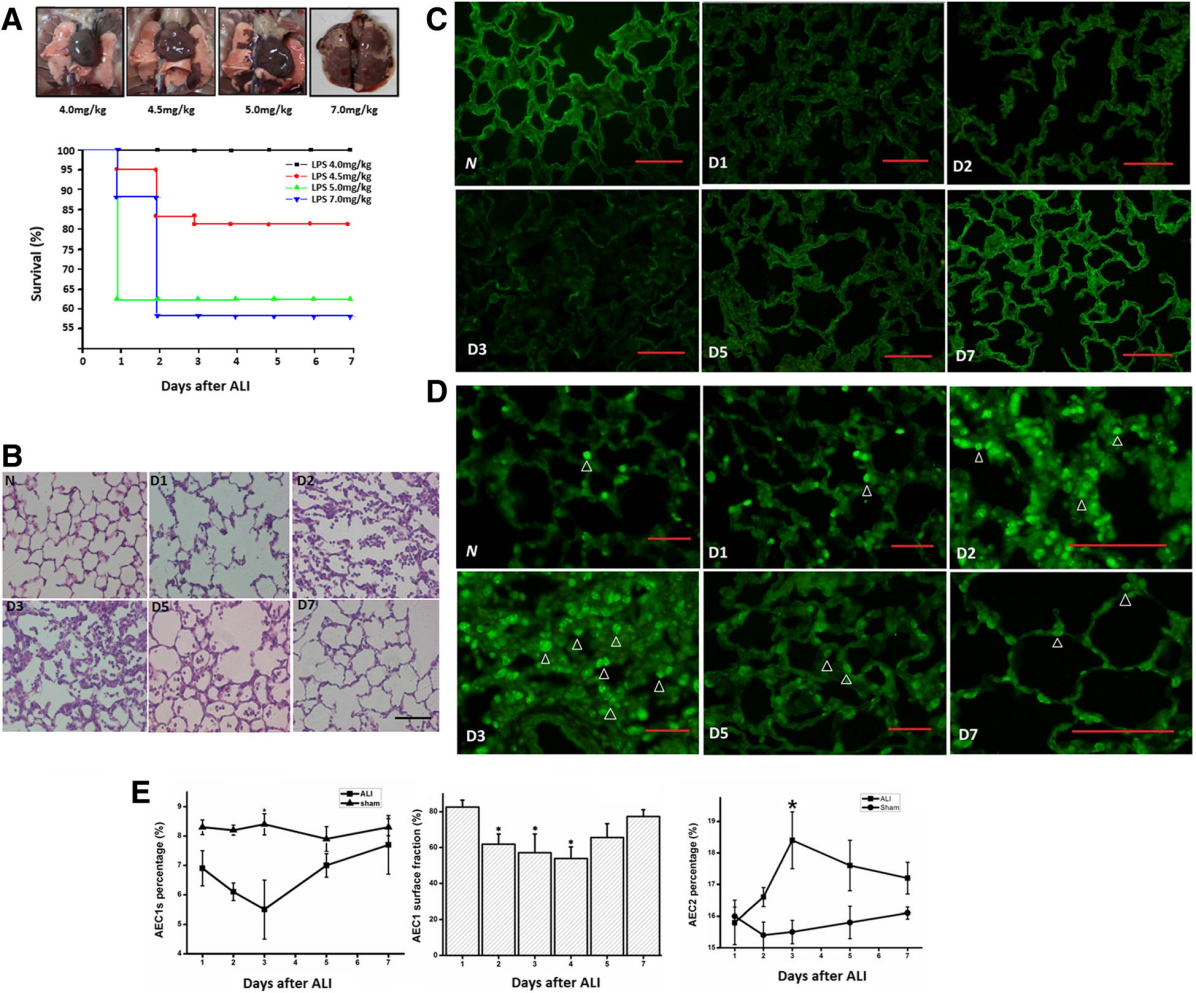
**a** The evaluation of severity of acute lung injury in different intratracheal injection of LPS concentrations. The lung imaging feature of four intratracheal injection LPS concentrations: 4.0, 4.5, 5.0 and 7.0 mg/kg, *n* = 20 for each group (*upper*). The survival rate of ALI rat of four intratracheal injection LPS concentrations (*lower*). **b** The hematoxylin and eosin stain of lungs after acute lung injury (200×). Most alveolar walls were fractured on the first day post injury (D1). On day 2 and 3, alveolar spaces were filled with a mixed neutrophilic and monocytic infiltrate, alveolar wall capillaries were congested. Alveolar hemorrhage was visible. Alveolar walls were lined with cuboidal epithelial cells which were proliferating AEC2s. On days 5 and 7, the formation of alveolar wall was recovered. Scale bar = 100 μm. **c** Immunofluorescence analysis of AEC1s in the rat lung after acute lung injury. Immunostaining for AQP5 (*green*) was performed on lung tissue from Normal (N), D1 (Day 1), D2 (Day 2), D3 (Day 3), D5 (Day 5) and D7 (Day7) days after injury. Scale bar = 100 μm. **d** The immunofluorescence analysis of AEC2s in rat lung after ALI. Immunostaining for pro-SPC (*green*) was performed on lung tissue from days 1, 2, 3, 5 and 7 after ALI. Arrowhead indicates proSPC positive AEC2s. Scale bar = 100 μm. **e** The cellular kinetics of AEC1s after ALI (The dose of LPS is 4.5 mg/kg in ALI rat model). Rat lung cell suspensions were incubation with anti-AQP5 antibody, the percentage of AQP5 positive cells was analyzed by flow cytometry analysis (*left*). *P = 0.026 (compared with sham group). The variance of percentage of covering surface of AEC1s after ALI (analyzed by Image J, Version 1.2). *P < 0.05 (compared with normal control) (*middle*). The flow cytometry analysis of AEC2s after acute lung injury. The rat whole lung cell suspensions were incubation with anti-proSPC antibody, the percentage of proSPC positive cells was analyzed. *P = 0.012 (*right*)

Rat hemorrhagic shock and LPS lung injury model was established, we found that rats exposed to 4.0 mg/kg LPS instilled intratracheally exhibited 100% survival, the area of pulmonary hemorrhage is only about 5%–10% (the hemorrhage area was quantified by HE staining and calculated by the proportion of hemorrhagic alveoli area). In contrast, the 7.0 mg/kg group exhibited 58% survival rate, the area of pulmonary hemorrhage is about 80–90% (Fig. 1a). We found 4.5 mg/kg LPS resulted in 80% survival rate, the area of pulmonary hemorrhage is about 30% (A mild to moderate acute lung injury which can stimulate the endogenous repair of lung tissue), so the proper LPS dose is 4.5 mg/kg.

#### Kinetics of AEC1s

Rat after ALI or sham surgery were sacrified on days 1, 2, 3, 5 and 7 after ALI to study time-specific proliferation of lung epithelial cells. According to the HE staining of rat lungs of different days after ALI, most alveolar walls are fractured on the first day after injury. On day 2 and 3, alveolar spaces are filled with a mixed neutrophilic and monocytic infiltrate, and alveolar wall capillaries are congested. Alveolar hemorrhage is visible. On days 5 and 7, the structure of alveolar wall is recovered (Fig. 1b).

The relative numbers of AEC1s were measured at various time points after acute lung injury using immunofluorescence and flow cytometry analysis. Immunofluorescence staining for AQP5 (AEC1 specific marker) showed that the first 3 days after ALI, the staining of AQP5 is discontinuous. A large number of AEC1s were fractured into fragments, and normal alveolar structure is destroyed. But from the 5th day, AEC1s began to repair and returned to a normal level on the 7th day (Fig. 1c). The flow cytometry analysis also showed that from the 5th day, the number of AEC1s gradually returned. On the 7th day the number returned to a normal level (Fig. 1e). Immunofluorescence staining for AQP5 was used to analyze the percentage of AEC1s covering the surface of alveoli (Fig. 1e), the result showed that, the kinetics of AEC1s was in concordance with the results of flow cytometry analysis, a decline on the first 3 days (About 60% of the normal level) and then gradually return to normal.

#### Kinetics of AEC2s

**Fig. 2:**
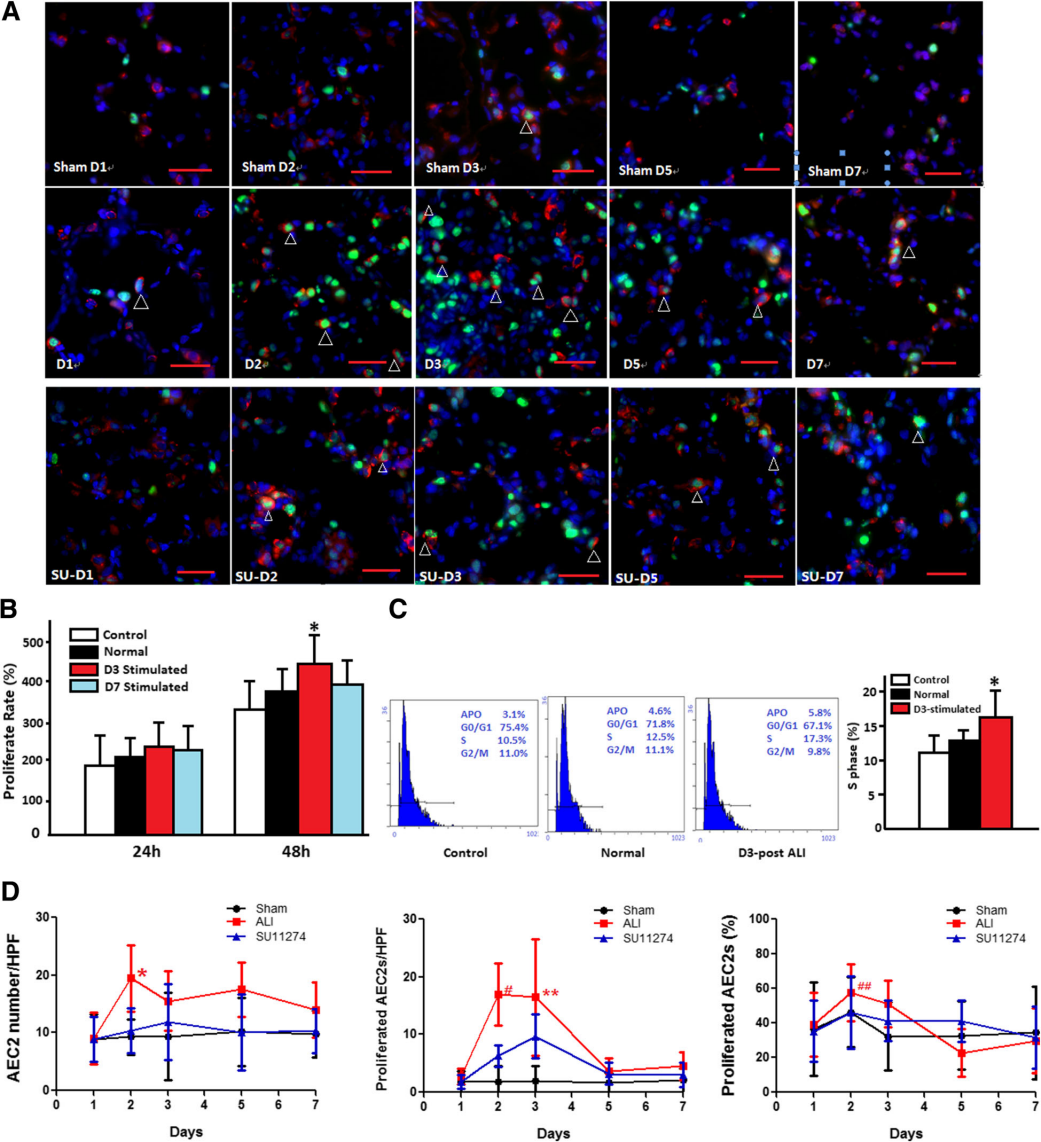
**a** The immunofluorescence of proliferated AEC2 cells after acute lung injury. Dual immunostaining (*arrowhead*) for proSPC (*red*) and PCNA (*green*) was used to monitor AEC2s proliferation in alveolar after ALI. Sham D1-D7: day 1 to day 7 post sham operation. D1-D7: day 1 to day 7 post acute lung injury. SU D1-D7: day 1 to day 7 post SU11274 treated. Scale bar = 100 μm. **b** The ALI milieu influenced the proliferation of AEC2s. The 3rd day after ALI’s lung tissue homogenate promotes the proliferation of AEC2s significantly, *P = 0.008 (Normal: co-cultured with normal rat LTH; D3: co-cultured with the 3rd day after ALI’s LTH; D7: co-cultured with the 7th day after ALI’s LTH). **c** The milieu of acute lung injury accelerates AEC2 cell cycle. The 3rd day after ALI’s lung tissue accelerates AEC2 cell cycle significantly (*P = 0.028). **d** Cellular kinetics of alveolar epithelial cells after acute lung injury. AEC2 numbers per high power field (HPF, 0.018 mm^2^) (*left*), proliferated AEC2s per HPF (*middle*) and the percentage of AEC2s (%) were indicated (*P = 0.009, **P =0.008 for the association of sham group vs. ALI group; ^#^ *P* = 0.031, ^##^ *P* =0.009 for the association of ALI group vs. SU11274 treated group)

Immunostaining for proSPC (AEC2 specific marker) was performed on lung tissue from 1, 2, 3, 5 and 7 days after injury (Fig. 1d) and AEC2s number after ALI were measured. There was an obvious increase of AEC2s number on the 2nd day, it reached a summit on the 3rd day and improved gradually after 5th day (Figs. 1d, 2d). The rat whole lung cell suspensions were incubated with FITC conjugated to anti-proSPC antibody, the percentage of AEC2s was analyzed by flow cytometry analysis (Fig. 1e), in sham-operated mice, there were no differences in numbers of AEC2s at various time points. However, the kinetics of AEC2s was in concordance with the results of quantitative analysis.

### AEC2s are damage resistant during acute lung injury

The relative numbers of proliferated cells and proliferated AEC2s were measured at different time points using immunofluorescence. Double-immunofluorescence staining for pro-SPC and Proliferating Cell Nuclear Antigen (PCNA) was used to monitor AEC2s proliferation after ALI. In sham group, there were no differences between proliferated cells and proliferated AEC2s on each day after acute lung injury. But there was a significant increase of proliferated cells and proliferated AEC2s after ALI, especially on the 2nd and 3rd day. Most alveolar walls were thickened, pulmonary alveoli were slightly shrinks, cubic or round proliferated AEC2s lined in the alveolar wall. On the 5th day to 7th day the number of proliferated cells and AEC2s returned to a normal level.

The results of quantitative stereological analysis showed that there was a significant increase in proliferated cells and proliferated AEC2s in ALI vs. sham was first detected on day 2 (29.5 ± 9.3 and 17.0 ± 5.4 cells per high power field), followed by further increases on day 3 (32.2 ± 17.4 and 16.5 ± 10.1 cells per high power field). The percentage of proliferated AEC2s increased to 57.6% on the 2nd day and 51.2% on the 3rd day. After a period of active lung regrowth (days 2–3), the percentage of AEC2s dropped to baseline levels by day 5–7 (Fig. 2d).

Previous studies from simian and rodent models suggested that AEC2s function as progenitor cells in the alveoli and proliferate and differentiate into AEC1s [14, 15]. Furthermore, Barkauskas et al. verified that AEC2s are stem cells in adult lung [11]. In our study, to determine whether acute lung injury affected the biological behavior of AEC2s, their number was quantitatively analyzed at various time points after ALI. Although significant AEC1s loss were observed by 48 h after ALI, the number of AEC2s did not significantly decrease at any time point. Immunofluorescence staining revealed that there was a significant increase of proliferated AEC2s in alveolar on day 2 and day 3 (Fig. 2a and d). Consistent with IF, FACS analysis showed that the abundance of AEC2s did not change during day 5 and day 7 (Fig. 1e). The result of HE staining also shows that alveolar walls are lined with cuboidal epithelial cells which are proliferating AEC2s on day 2 and day 3. On day 5 and 7, the structure of alveolar wall recovered. These results proved that AEC2s are damage resistant during acute lung injury, and they might be the main cell involved in lung injury and repair.

#### The milieu of acute lung injury promoted AEC2s growth and accelerated AEC2s cell cycle

We obtained bronchoalveolar lavage fluid (BAL) and lung tissue homogenate (LTH) from acute lung injury rats, to represent the acute lung injury in vivo damage environment, and compare their effects with BAL and LTH from normal rats when added to primary cultured, attached, non-confluent AEC2s (attached for 48 h).

AEC2s were isolated by immunomagnetic separation. After the preparation of rat lung single cell suspension, rat IgG panning was used to deplete immunocytes expressing FcR to enrich for alveolar epithelial cells. T1α-pos cells (AEC1s) (Purity 90 ± 4%) were firstly separated from rat lung single cell suspension, then EpCAM-pos cells (AEC2s) were isolated (Purity 86 ± 5%). Freshly isolated AEC2s are essentially non-proliferative, with greater than 90% of the population in G1 phase of the cell cycle, and remain quiescent in culture [16, 17]. The 3rd day’s damaged Lung tissue homogenates (100ug/ml) were added to 48-h cultured, adherent AEC2s. The BAL had no effect on the proliferation of AEC2s. But the 3rd day's acute lung LTH significantly increased the cell numbers when stimulated for 48 h (*P* = 0.008, Fig. 2b) and more AEC2s were in the S phase of cell cycle (Fig. 2c). The cell cycle distribution was measured using propidium iodide (PI) staining and detected by flow cytometry assay. The proportion of AEC2s in S phase of cell cycle stimulated with 3rd day’s LTH was approximately 19.3 ± 2.1%, while it was only 14.0 ± 2.6% of AEC2s stimulated with Normal lung LTH (Fig. 2c), suggesting that the milieu of acute lung injury is conducive to AEC2 growth. In contrast, normal lung LTH and the 7th day’s LTH did not significantly affect AEC2 proliferation (Fig. 2b).

### The molecular mechanism of acute lung injury milieu induced AEC2 proliferation

#### Increased HGF level are detected in in acute lung injury milieu

**Fig. 3:**
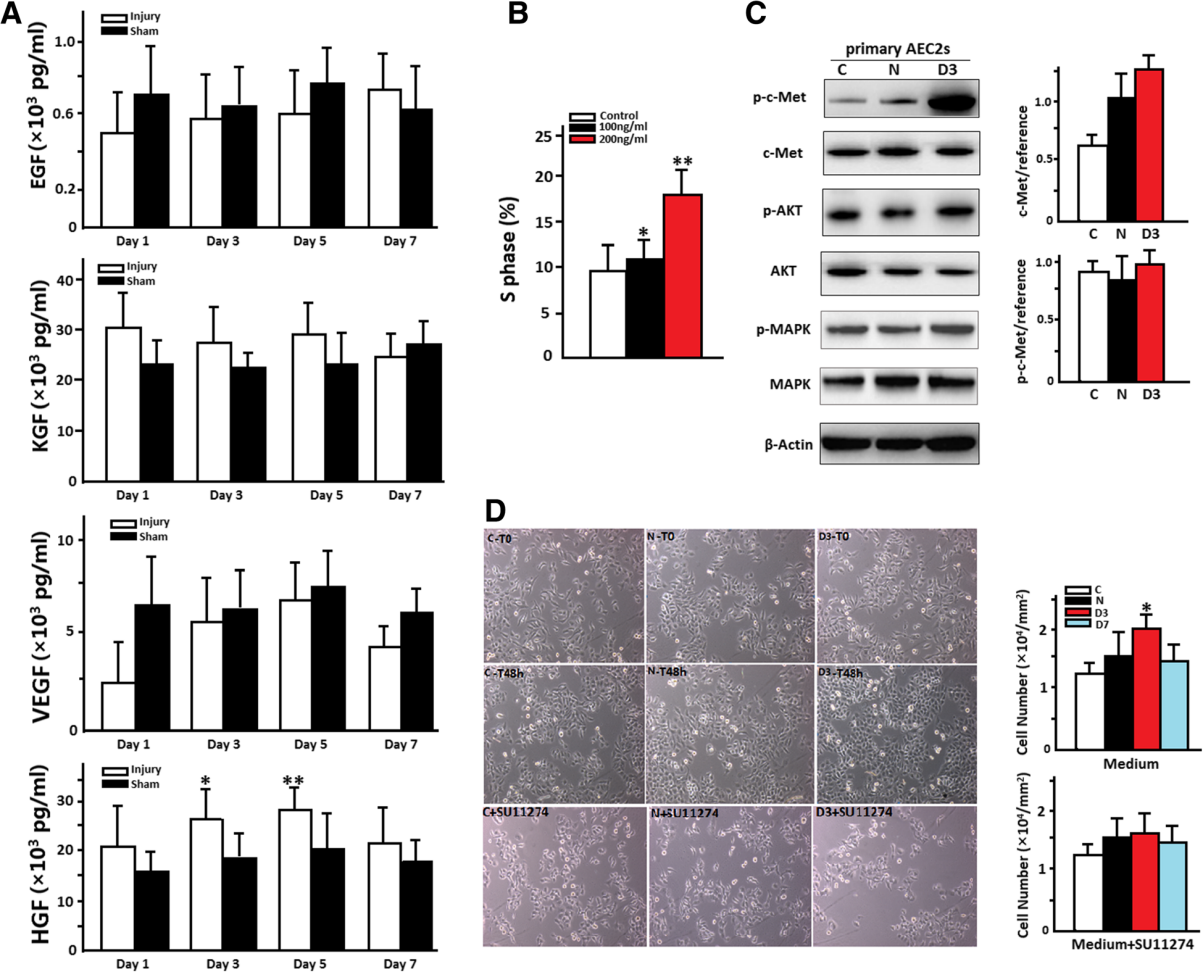
**a** The concentration of HGF, KGF, VEGF and EGF in rat lung homogenate at different days post injury using enzyme linked immunosorbent assay (ELISA). *P = 0.028, **P = 0.013. **b** HGF accelerates AEC2 cell cycle in vitro. *P = 0.042, **P = 0.011. **c** The effect of ALI on the expression of key protein of the HGF/c-Met signaling. Image-J was used in the comparing of the intensity of bands of Western Blot. The 3rd day after ALI’s LTH can promote the significant higher expression of p-c-Met, *p = 0.018. **d** The effect of the 3rd day after ALI’s LTH on the proliferation of AEC2’s in vitro. AEC2’s had an increased proliferation rate when co-cultured with the 3rd day after ALI’s LTH (100ug/ml, 48 h). When co-cultured with SU11274, the proliferation rate of AEC2s co-cultured with the 3rd day after ALI’s LTH was inhibited (*left*). The quantitative analysis of AEC2 proliferation with or without SU11274 (*right*), *P = 0.004

To observe important cytokines’ expression in lung, we detected VEGF, EGF, KGF and HGF in lung homogenate, and found that HGF was the only cytokine significantly elevated after acute lung injury, at levels >1.5-fold higher than sham control on the 2nd and 3rd day after acute lung injury (Fig. 3b), although there was not a significant difference by the first day post injury. We next examined whether HGF can accelerate AEC2 cell cycle in vitro. Figure 3c presents the cell cycle analysis of primary AEC2s and HGF stimulated AEC2s in vitro. 100 and 200 ng/ml HGF can significantly accelerate AEC2 cell cycle (the percentage of AEC2s in S phase increased significantly), indicating that the proliferation of AEC2 after acute lung injury may be induced by the elevated HGF.

#### The activation of HGF/c-Met signaling pathway and its contribution to alveolar type II cell proliferation after acute lung injury in vitro and in vivo

To clearly establish the relationship between HGF/c-Met activation and AEC2s proliferation, we observed the expression level of the key proteins. Immuno-magnetic separated AEC2s were co-cultured with normal or the 3rd day’s milieu of ALI in vitro. We found that the 3rd day’s milieu of ALI can induce the significant higher expression of phospho-c-Met (p-c-Met) while there is no difference of the expression of total c-Met (*p* = 0.018, Fig. 3d), which indicated that the regulation of high expression of p-c-Met was not due to the high expression of total c-Met. SU11274 is a selective, ATP-competitive inhibitor of MET receptor tyrosine kinase, it reduced the 3rd day ALI’s Lung tissue homogenates-increased AEC2s cell proliferation significantly (*P* = 0.004) (Fig. 3e). Other important tyrosine kinase pathways (AKT and MAPK) had been detected, but there was no difference when treated with the 3rd day ALI’s Lung tissue homogenates (Fig. 3d).

Furthermore, in order to observe the function of HGF/c-Met signaling pathway in the proliferation of AEC2s in vivo, we treated rats with SU11274 every day after ALI. The results of quantitative stereological analysis showed that the proliferated AEC2s number was 6.3 ± 1.9 per high power field after SU11274 treated, while it is 17.0 ± 5.4 in ALI group on the 2nd day post ALI (^#^ *P* = 0.031, Fig. 2d). The percentage of proliferated AEC2s is also lower after SU11274 treated on the 2nd day post ALI (^##^ *P* = 0.009, Fig. 2d).

## Discussion

In the rodent lung, there are several stem cell niches that are the key in maintaining the epithelial layers of lung tissue. Kim and colleagues describe a niche in the bronchioalveolar duct junction of adult mouse lung, which enriched, propagated, and differentiated these stem cells in vitro [5]. Kumar et al. infect the mouse airways with influenza A (H1N1) virus, they found p63+ Krt5+ basal-like cells expanded locally, organized into growing spheres with a lumen, and subsequently assumed expression of alveolar specific proteins, all indicative of proper differentiation and regeneration in vivo [1]. Vaughan et al. proposed that rare LNEPs residing in the bronchiolar airways are activated after influenza virus infection to expand and give rise to induced Krt-5+ cells in the alveolar parenchyma and more differentiated lineage-committed bronchiolar and alveolar epithelial cells [7]. Desai et al. showed that, during development, although AT1 and AT2 cells arisen directly from a bipotent progenitor, after birth of new AT1 cells derived from rare, self-renewing, long-lived, mature AT2 cells that produce slowly expanding clonal foci of alveolar renewal [18]. These stem cells are all rare cells (from 0.1 to 0.01%) and play their role in regeneration through differentiation into AECs, especially AEC2s.

Many lineage-tracing experiments had established that SPC+ AEC2s can proliferate and give rise to AEC1s in vivo [12]. Furthermore, new evidence shows that the same SPC+ cells can maintain the AEC2 population over the long term, an important criterion for defining the population as containing stem cells. In the unperturbed lung, lineage-labeled AEC2s give rise to only small clones of daughter cells, and there is a low rate of differentiation into AEC1s [19]. But there is hardly any study examined the cellular kinetics of AEC1s and AEC2s post acute lung injury systematicly. Using the hemorrhagic shock and LPS lung injury rat model, we found that the number of AEC1s began to decline on the 1st day and declined to a bottom on the 3rd day post injury, then gradually returned to a normal level. The kinetics of AEC2s was different to AEC1s, a significant increase in AEC2s number was detected on the 3rd day, followed by a further increase from the 5th day to 7th day, then AEC2 numbers gradually returned to normal. Double-immunofluorescence staining for pro-SPC and PCNA showed that the percentage of proliferated AEC2s was significantly increased on the 3rd day. After a period of active lung repair, AEC2 numbers dropped to baseline levels by day 7. It means that AEC2 significantly proliferated after acute lung injury. They are injury resistant cells in acute lung injury and may have important roles in the regeneration of damaged lung.

The local environment of a cell dictates cell fate, and bioengineering of complex organs like the lung will require a detailed knowledge of regional microenvironments to faithfully recapitulate regeneration. Similarly, the effectiveness of stem cell therapy to ameliorate tissue damage could likely be optimized if the specific damage niche was well characterized. After acute lung injury, the milieu of the damaged tissue will determine the rate and nature of alveolar epithelial repair and is therefore of great interest. We speculate that the milieu of acute lung injury can promote the proliferation of AEC2s due to the cytokines released so we observed whether the milieu of acute lung injury had biological effect on AEC2s. The cytokine release of the lung homogenate was investigated. HGF was the only cytokine significantly elevated on the 2nd and 3rd day after acute lung injury. It can significantly accelerate AEC2 cell cycle in vitro, indicating that the proliferation of AEC2 after acute lung injury may be induced by the elevated HGF.

HGF is a potent mitogen and motogen for various epithelial cells [20, 21]. It is produced following HCl- or bleomycin-induced acute lung injury and plays a role in pulmonary epithelial cell regeneration [22, 23]. Studies have also demonstrated that HGF is responsible for most growth-promoting activity for AEC2s cells, compared with EGF, TGF-a, acidic fibroblast growth factor, and keratinocyte growth factor [24]. All biological effects of HGF are mediated by a single tyrosine kinase receptor (c-Met), which is mainly expressed in cells with an epithelial or endothelial origin [24, 25]. Incubation with HGF results in the activation of c-Met and increase of proliferation in lung adenocarcinoma cells and isolated type II cells and stimulation of ERK1/2 phosphorylation in lung adenocarcinoma cells [26]. To clearly establish the relationship between HGF/c-Met activation and AEC2 proliferation, we observed the key proteins’ expression. We found that the 3rd day’s milieu of ALI can promote significant higher expression of p-c-Met while there is no difference of the expression of total c-Met. The Met kinase inhibitor SU11274 significantly reduced 3rd day’s damaged Lung tissue homogenates-increased AEC2s cell proliferation. Furthermore, we verified the role of c-Met in the proliferation of AEC2s in vivo by treated of c-Met inhibitor SU11274. HGF/c-Met signaling is likely a major factor responsible for the pulmonary epithelial cell proliferation after acute lung injury.

## Conclusion

In conclusion, our present findings support the hypothesis that the milieu of acute lung injury can stimulate pulmonary epithelial cell proliferation after acute lung injury. The HGF/c-Met signaling is likely a major factor responsible for the pulmonary epithelial cell proliferation. These data have significant implications with regard to the role of HGF/c-Met signaling in injury repair. Extension of this work into the regulation of growth factor balance is required for alveolar epithelial cell maintenance and repair could have important translational clinical and bioengineering applications for patients with alveolar damage or disease.

## Abbreviations

ABB: Air-blood barrier
AEC1: Type 1 alveolar epithelial cells
AEC2: Type 2 alveolar epithelial cells
ALI: Acute Lung Injury
AQP5: Aquaporin 5
BAL: Bronchoalveolar lavage fluid
DASCs: Distal airway stem cells
HGF: Hepatocyte growth factor
LNEP: Lineage-negative epithelial progenitor
LPS: Lipopolysaccharide
LTH: Lung tissue homogenate
MLSCs: Lung multipotent stem cells
PCNA: Proliferating cell nuclear antigen
